# Universal access to surgical care—A global public health priority

**DOI:** 10.1371/journal.pgph.0004326

**Published:** 2025-04-09

**Authors:** Salome Maswime, Sudha Jayaraman, Olufunke Alaba, Magda Robalo

**Affiliations:** 1 Global Surgery Division, Department of Surgery, Faculty of Health Sciences, University of Cape Town, Cape Town, South Africa; 2 Department of Surgery, Center for Global Surgery, University of Utah, Salt Lake City, Utah, United States of America; 3 Health Economics Division, School of Public Health, University of Cape Town, Cape Town, South Africa; 4 Institute for Global Health and Development, Bissau, Guinea-Bissau; PLOS: Public Library of Science, UNITED STATES OF AMERICA

## Introduction

Universal health coverage aims to ensure that all people have access to the full range of quality health services when and where they need them, without financial hardship. This includes the full range of services, including preventative, curative, rehabilitative and palliative care [1]. Despite advances in medical care, and progress in improving access to health care, it is estimated that 5 billion people, over half of the world’s population, do not have access to safe and timely surgery when required. The Lancet Commission on Global Surgery in 2015 described the lack of access to surgery as a major public health concern [2].

## Global surgery

Global surgery is a relatively new and exciting multidisciplinary field of enquiry, research, practice, and advocacy that aims to improve health outcomes and achieve health equity for all people who need surgical, obstetric and anaesthesia care, with a special emphasis on underserved, marginalized populations and those in humanitarian crisis [3]. As an emerging academic discipline responding to a major unmet need in population health, there is an evolving demand for formal teaching, training and learning in global surgery, not only for trainees in surgical disciplines but also for trainees from public and primary healthcare disciplines who are interested in and those who work in the provision of surgery care.

## A global strategy for operative care

In 2015 the World Health Assembly passed a resolution (WHA 68.15) that framed emergency and essential surgical and anaesthesia care as essential components of Universal Health Coverage [4]. As the world emerged from the COVID-19 pandemic, in 2023, the 76th World Health Assembly refocused on surgery and anaesthesia with resolution WHA76.2, integrated emergency, critical and operative care for universal health coverage and protection from health emergencies. The resolution aimed to enhance capacity at all levels, and support for the efforts of WHO Member States and other relevant actors to strengthen the delivery of emergency, critical and operative care, including health emergency preparedness, readiness, response and recovery, across the spectrum of health services [5]. In support of resolution 76.2, in 2024 the 77th World Health Assembly passed a decision to develop a Global Strategy and Action Plan for Integrated Emergency, Critical and Operative care for 2026-2035. The Global Strategy will establish the high-level vision, goals, and strategic objectives for emergency, critical and operative care (ECO) [6].

## Public health and access to surgery

In the last decade, Global Surgery efforts have evolved from primarily short-term mission trips and capacity building programmes, to sustainable health system strengthening approaches [7]. Surgical providers and governments in low-and middle-income countries have developed programs, plans and policies to improve access to surgery. Ma et al wrote about Global Surgery accelerating the shift from the focus on patient-centred technological innovations such as robots and minimally invasive surgery, to systems strengthening innovations that provide the infrastructure for functional surgical services and systems in poorly resourced settings [8]. Despite the efforts, the co-ordination between global surgery experts and public health organisations has not gained traction. Global Surgery remains, perhaps, not a step-child in global health anymore, but a topic that seldom makes it to global public health classes, discussions, meetings or conferences.

The complexity of improving access to surgery in a world that is struggling to provide access to basic healthcare to underserved communities, amid pandemics and geopolitical crisis, understandably overwhelms national and global health leaders, who are battling to provide healthcare services in countries facing basic challenges like providing access to water, electricity, oxygen, internet, medicines, contraceptives or childhood vaccinations. Despite the unmet need for surgery, the surgical burden of disease remains high, and every country needs to address the need for surgery as a public health priority to be able to maximize potential for economic growth. This approach is essential for addressing the significant burden of surgically treatable diseases, which contribute to 11% to 15% of health impairments globally [9]. It was estimated that 18 million deaths are preventable each year from Surgery, Anaesthesia and Obstetric care [2]

## Proposed public health strategies to improve access to surgical care

(refer to Fig 1)

**Fig 1:**
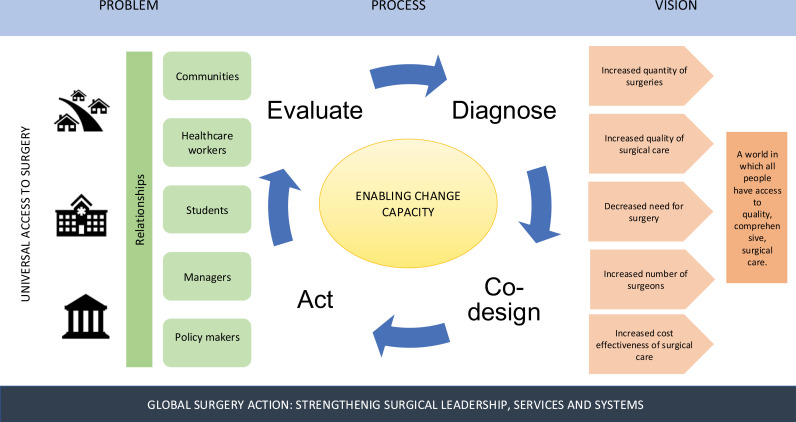
Global Surgery in Action: strengthening systems and services, supporting leadership and governance.

1. Increase the number of people with access to essential surgical operations

Surgical missions and outreach programmes, often led by donor-funded programmes, have been used by many countries to provide surgical operations that they would not ordinarily be able to provide in low- and middle-income countries. These programmes should not depend only on external partners, they should be determined by local leaders based on the burden of disease, with capacity building, integration and sustainability measures in place and incorporated into the national surgical programmes.

2. Increase the number of the surgical workforce

The surgical workforce indicates what types of surgeries can be done by countries. It, however, often does not correlate with the surgical burden of disease. Prioritisation on which surgical specialisations countries need to focus on is paramount. Training needs to be based on the unmet need for surgery, with emphasis on disciplines that enable surgery to happen, such as nursing and anaesthesia care, and disciplines that support the rehabilitation of surgical patients after surgery. Apart from training, there is a need to put into place retention strategies that ensure that skilled personnel are retained where they are needed, including in rural areas.

3. Reduce the need for surgery

Much emphasis has been placed on increasing the number of surgeries to close the gap in unmet needs. Yet, public health often focuses on strategies to prevent disease at primary levels of care. Population wide strategies to prevent surgical diseases and trauma would go a long way towards reducing the need for surgery. The engagement of communities and the training of community health workers on prevention and care of surgical patients at home; as well as policy engagement to legislate programmes that will reduce the surgical burden of disease, would contribute to addressing this important public health concern.

4. Improve the quality of surgery and the health system

According to the African Surgical Outcomes Study, Africans were twice more likely to die from complications of surgery than people in high income countries [10]. Surgical complications contribute to the perioperative mortality, especially in countries whose health systems are not able to provide critical services such as blood banks, intensive care units, ventilators, efficient referral systems and multidisciplinary care. Addressing failure to rescue, or preparedness for health systems to address surgical complications is important to strengthen functional health systems. Ensuring that health facilities are equipped with essential surgical tools and maintaining high hygiene standards are imperative to successful surgery.

5. Improve cost-effective forms of surgery and surgical innovation

Worldwide, 81.3 million people incur catastrophic expenditures related to surgical healthcare each year [11]. In countries where patients must pay to access basic health care services, they are sometimes forced to forego or delay surgery for as long as possible, considering the financial implications that it may have on their financial wellbeing. Delays to surgery can advance diseases that may become incurable, while prolonging disability and lack of function or causing premature mortality. It is therefore critical to ensure that countries have in place financial schemes that prevent patient’s financial hardship due to diseases that need surgical care. Financial protection policies that protect patients from catastrophic health expenditures related to surgical care are important to improve and increase access. Investment in the development and utilization of cost-effective technologies, including telehealth, can significantly enhance healthcare access and efficiency, particularly among the low-income populations. This can bridge the service delivery gap.

## Conclusion

Investment in surgery not only saves more lives but also contributes to healthier populations, and enhances economic growth and prosperity, which in turn contribute to improvs the global economy and reduces disparities. Effective public health strategies to improve access to surgery will ensure we are on our way to achieving universal health coverage by 2030.
